# Scaling nitrogen and carbon interactions: what are the consequences of biological buffering?

**DOI:** 10.1002/ece3.1565

**Published:** 2015-06-25

**Authors:** David J Weston, Alistair Rogers, Timothy J Tschaplinski, Lee E Gunter, Sara A Jawdy, Nancy L Engle, Lindsey E Heady, Gerald A Tuskan, Stan D Wullschleger

**Affiliations:** 1 Biosciences Division, Oak Ridge National LaboratoryOak Ridge, Tennessee, 37831-6407; 2 Biological, Environmental & Climate Sciences Department, Brookhaven National LaboratoryUpton, New York, 11973-5000; 3 Environmental Sciences Division, Oak Ridge National LaboratoryOak Ridge, Tennessee, 37831-6301

**Keywords:** Buffering, carbon, ecological genomics, networks, nitrogen, robustness, scaling

## Abstract

Understanding the consequences of elevated CO_2_ (eCO_2_; 800 ppm) on terrestrial ecosystems is a central theme in global change biology, but relatively little is known about how altered plant C and N metabolism influences higher levels of biological organization. Here, we investigate the consequences of C and N interactions by genetically modifying the N-assimilation pathway in *Arabidopsis* and initiating growth chamber and mesocosm competition studies at current CO_2_ (cCO_2_; 400 ppm) and eCO_2_ over multiple generations. Using a suite of ecological, physiological, and molecular genomic tools, we show that a single-gene mutant of a key enzyme (*nia2*) elicited a highly orchestrated buffering response starting with a fivefold increase in the expression of a gene paralog (*nia1*) and a 63% increase in the expression of gene network module enriched for N-assimilation genes. The genetic perturbation reduced amino acids, protein, and TCA-cycle intermediate concentrations in the *nia2* mutant compared to the wild-type, while eCO_2_ mainly increased carbohydrate concentrations. The mutant had reduced net photosynthetic rates due to a 27% decrease in carboxylation capacity and an 18% decrease in electron transport rates. The expression of these buffering mechanisms resulted in a penalty that negatively correlated with fitness and population dynamics yet showed only minor alterations in our estimates of population function, including total per unit area biomass, ground cover, and leaf area index. This study provides insight into the consequences of buffering mechanisms that occur post-genetic perturbations in the N pathway and the associated outcomes these buffering systems have on plant populations relative to eCO_2_.

## Introduction

Hierarchical theory states that functional importance is observed at higher levels of biological organization, while lower levels explain mechanism (Bartholomew 1964). In regard to carbon (C) and nitrogen (N) interactions, metabolic processes at lower levels of organization [e.g., at the DNA level] elicit complex regulatory networks that scale through organelles, cells, and tissues to influence organismal physiology, developmental ontogeny, and fitness (Reich et al. 2006a,b). Complicating this hierarchical view is that each level of biological organization is inherently robust against perturbation (Dunne et al. 2002; Melian and Bascompte 2002; Delattre and Felix 2009; Greenbury et al. 2010). Here, we use Masel and Siegal (2009) definition of “robustness,” where the system produces a relatively invariant set of outputs regardless of genetic and environmental perturbation. So, it remains an open question as to whether perturbations at lower levels of organization will cascade and/or intensify to influence higher organizational levels or whether inherent buffering mechanisms will mitigate potential effects from genetic and environmental perturbation.

To address this question, we must first have a basic understanding of (1) C and N metabolic pathways; (2) how changes in these pathways alter whole-plant physiology and development; and (3) the mechanisms by which buffering occurs to mitigate the effects of metabolic perturbations on these organismal processes. At the metabolic level, C and N interactions begin with amino acids biosynthesis. This process requires inorganic N, C-skeletons, reducing equivalents and ATP derived from photosynthesis or carbohydrate breakdown (reviewed in Nunes-Nesi et al. 2010)). This metabolic codependence requires pathway cross-talk that has been demonstrated in a number of molecular-based studies where the feeding of sugars induces the expression of numerous genes involved in N metabolism (Hoff et al. 1994; Lejay et al. 1999). Likewise, application of nitrate induced the expression of genes encoding enzymes important for C metabolism including glycolysis pathway (Wang et al. 2003). Together, C, N, or C-N interaction controls nearly half of the expressed transcripts in the model plant *Arabidopsis thaliana* (Gutierrez et al. 2007).

These regulatory networks extent organization from molecular, organelle, cell, and tissue levels to influence organism-level process such as N-uptake, N-allocation, and carbon assimilation rate. By way of relative importance, N is in greater demand by plants than any other mineral due to the large amount of N invested in the photosynthetic apparatus, especially in Rubisco that can account for up to 25% of leaf N (Sage et al. 1987). Plants need to balance the investment of N-containing proteins among different biological processes including photosynthesis, respiration, growth, and defense (Chapin et al. 1990; Evans and Poorter 2001; Xu et al. 2012). These processes, in turn, influence developmental programs including root architecture, flowering, and root:shoot ratio (Norby et al. 1999; Pritchard et al. 1999; Kinugasa et al. 2003), thereby influencing population dynamics and perhaps ecosystem-level responses (e.g., net ecosystem exchange, NEE).

Each of these levels is, however, permeated with some level of robustness to genetic and environmental perturbation through mechanisms that are not fully understood or completely defined. Despite its putative consequences, the study of robustness is often conducted within the narrow view of the discipline of the investigator (e.g., cells and tissue for molecular biologists, organism, and above for ecologists).

Here, we investigated C and N interactions through multiple levels of biological organization and measure the consequences of the perturbed state relative to control states within a molecular ecology context (Reusch and Wood 2007) by knocking down a key gene in the *Arabidopsis thaliana* nitrogen assimilation pathway and scaling that response from gene to pathway to organism to population under eCO_2_. Specifically, we test the hypotheses that (1) buffering mechanisms will cascade through levels of biological organization and influence population dynamics; and (2) eCO_2_ will interact with our genetic perturbation to further offset C and N metabolism to decrease fitness of the mutant and alter population dynamics and function.

## Materials and Methods

### Experimental design and sampling

Two experimental strategies were used to investigate the impact of a genetic perturbation to a key gene in the *Arabidopsis thaliana* nitrogen assimilation pathway and scaling that response from gene to pathway to organism to population under eCO_2_. Soil-based mesocosms constructed in the greenhouse and described below allowed for wild-type and *nia2* mutants to be grown in competitive mixtures over multiple generations. Specifically, the *nia2* mutant is a T-DNA insertion within the At1g37130 allele (SALK_138297C) and confirmed for reduced nitrate reductase activity that is the main entry point for plant inorganic N metabolism. Hydroponic mesocosms in the greenhouse enabled a more in-depth, yet complementary focus on the biochemical and photosynthetic differences between wild-type and *nia2* mutants. Together, these two approaches contribute to a molecular ecosystem perspective on “robustness” as defined by Masel and Siegal (2009).

Soil-based mesocosms: The 36 mesocosms used in this experiment consisted of a stainless-steel soil bin and a transparent aboveground chamber. The soil bin (50 × 50 × 30 cm) was filled with an artificial mixture of one part sandy loam and four parts washed river sand. Each soil bin was equipped with a 50 × 50 × 45 cm clear acrylic plastic (i.e., Lucite) enclosure designed to allow easy access to plants for sampling and to facilitate exposing plants to ambient (430 ± 50 ppm) and elevated (810 ± 50 ppm) CO_2_ concentrations. Air exiting the mesocosms was routed to an infrared gas analyzer (Li-820; Li-Cor, Inc., Lincoln, NE) where absolute CO_2_ concentration was monitored. Mesocosms were instrument with multiple environmental sensors including air and soil temperature, soil water content, relative humidity, and photosynthetically active radiation (i.e., PAR). All data were collected as hourly means and logged on a Campbell Scientific CR10× data logger (Campbell Scientific, Inc., Logan, UT). A drip-irrigation system was included within each mesocosm.

Six monocultures of *Arabidopsis thaliana* “Columbia”- wild-type plants, six monocultures of a mutant, single-gene T-DNA insertion variant in the *Nia2* allele (At1g37130; gifted from Nigel Crawford Lab, UC San Diego, CA), and six mixtures with equal density plantings of each genotype were established within designated mesocosm chambers and exposed to ambient (430 ± 50 ppm) and elevated (810 ± 50 ppm) [CO_2_]. This resulted in 36 total mesocosms (three genotype conditions × 6 replicates × 2 [CO_2_]). The 36 mesocosms were distributed across six benches in a temperature-controlled greenhouse and arranged in a randomized completed block design with six replicates. Sample collection in relation to development and environmental conditions was previously described (May et al. 2013). In brief, samples were collected for leaves of at least 100 plants per mesocosm using a random assignment through a point quadrant system. Temperature was maintained between 24 and 18°C (day/night); natural lighting was augmented with sodium halide lamps on overcast days; and humidity was maintained at natural conditions. After self-seeding, watering was turned off for 2 weeks to synchronize the next generation start. Flowering was initiated within 5–6 weeks with inflorescence stems observed around the 70th day after plant germination. Plants grown at ambient [CO_2_] flowered about a week later than those grown at elevated [CO_2_]. The leaves were pooled and immediately flash frozen in liquid N_2_.

Hydroponic mesocosms: Seed of the wild-type and *nia2* mutant was germinated and plants grown in hydroponic culture following a modification of the procedure previously described (Noren et al. 2004). Seeds were sterilized in 70% ethanol for 5 min, agitated in 0.5% (w/v) sodium dodecyl sulfate solution for 15 min, and triple rinsed in sterile water. Seeds were stratified in the dark at 4°C for 2 to 3 day. Stratified seeds were suspended in a 0.1% phytagar solution and dispensed onto pipette tips containing sterile nutrient agar. Once seeds germinated, pipettes containing seedlings were transferred to large (56 × 56 × 18 cm) ebb and flow hydroponic systems (American Agritech, Tempe, AZ) consisting of a reservoir containing the nutrient solution with a circulating pump and constant aeration from aquarium stones attached to a four-channel air pump. Twenty hydroponic systems were used in these experiments with 10 systems allocated to each of two walk-in Conviron BWD80 growth rooms (Winnipeg, Manitoba, Canada). Each system contained eight wild-type and eight *nia2* mutant seedlings. One growth room was maintained at ambient (400 *μ*mol·mol^−1^), and the other was maintained at twice ambient or elevated (800 *μ*mol·mol^−1^) CO_2_ concentration. Carbon dioxide was supplied via injection from a liquid storage tank located on site. A control system was implemented to monitor CO_2_ concentrations within each growth room using an infrared gas analyzer (LI-820; LiCor), which sampled air concentrations every 2 min. The environmental conditions within each growth room were maintained at a day/night temperature of 22/18°C, an 8-h photoperiod, and 80% relative humidity. Experiments were repeated twice with CO_2_ treatments switched between growth rooms. Experimental design was a 2 × 2 factorial with six replicates.

### Enzyme activities, transcript, and metabolite profiles

The randomly collected leaf tissue (1–2 g) was ground using motor and pestle with liquid N_2_ and divided into three aliquots corresponding to transcript, enzyme, and metabolite assays. For transcript profiling, microarrays were printed with Operon v.3.1 (Eurofins MWG Operon LLC, Huntsville, AL, USA), 70-mer oligos obtained from the Galbraith laboratory. RNA was isolated, labeled, hybridized, scanned, and data normalized as previously described (Weston et al. 2008), and raw data are deposited at NCBI GEO (GSE16068). Determination of differentially expressed genes was as described in (Weston et al. 2011) using the R statistical language and Bioconductor packages (Smyth 2005).

Gas chromatography–mass spectrometry (GC-MS) was used to quantify the relative concentrations (ug sorbitol equivalents/g fresh weight (FW)) of target metabolites. Analyses were performed on leaves (∼0.05 g FW) that were immediately frozen and then ground in liquid N_2_ and stored at −80°C until extraction prior to trimethylsilyl (TMS) derivatization and analysis. Sample preparation was as described elsewhere (Morse et al. 2007). After 2 day, 2-*μ*L aliquots were injected into a DSQII (Thermo Fisher Scientific, Waltham, MA) GC-MS, fitted with an Rtx-5MS (cross-linked 5% PH ME Siloxane) 30 m × 0.25 mm × 0.25 *μ*m film thickness capillary column (Restek, Bellefonte, PA). The standard quadrupole GC-MS was operated in electron impact (70 eV) ionization mode, with 6 full-spectrum (70–650 Da) scans per second. Gas (helium) flow was set at 1.1 mL·min^−1^ with in injection port configured in the splitless mode. The injection port and detector temperatures were set to 220 and 300°C, respectively. The initial oven temperature was held at 50°C for 2 min and was programmed to increase at 20°C per min to 325°C and held for another 11.25 min, before cycling back to the initial conditions. The target metabolites were integrated using a key selected ion (and confirmed by three additional characteristic m/z fragments), rather than the total ion current, to minimize integrating co-eluting metabolites. Peaks were quantified by area integration and the concentrations were normalized to the quantity of the internal standard (sorbitol) recovered, amount of sample extracted, derivatized, and injected. Starch, free amino acid, and protein content were estimated as described previously (Ainsworth et al. 2007).

Enzyme activities were assayed using a 96-head liquid handling robot (Evolution P3; PerkinElmer, Wellesey, MA) to perform stopped and cycling assays as previously described (Gibon et al. 2004). Maximum nitrate reductase and glutamine synthetase activities were determined using end-point assays (Gibon et al. 2004; Gillespie et al. 2011). Total free amino acid content was determined using a fluorogenic-based microplate assay (Bantan-Polak et al. 2001).

### Physiology and population-level measurements

#### Gas exchange methods

Transient decreases in chloroplast inorganic phosphate concentration and photosystem II efficiency may alter the response of photosynthesis (*A*) to [CO_2_] and can occur within a few hours of illumination (Bernacchi et al. 2005). To avoid this complication, plants were kept in the dark on the day of measurement. Approximately 30 min prior to measurement, individual plants were transferred from the hydroponics systems into test tubes filled with water and acclimated to light under a fluorescent light bank (350 *μ*mol·m^−2^·sec^−1^). Gas exchange measurements were made using four LI-6400 gas exchange systems (LI-COR) equipped with 6-cm^2^ leaf chambers. Leaf chamber temperature was maintained at 25°C for all measurements using the Peltier-based temperature control. Following analysis of the response of *A* to PPFD, light levels were maintained at a saturating 1000 *μ*mol·m^−2^·sec^−1^ using the leaf chambers integrated red-blue light source. Air entering the gas exchange system was humidified by adding ca. 3 mL of water to the soda lime as per manufacturer’s instructions. This was required to maintain a vapor pressure deficit <1.1 kPa.

Plants were randomly assigned to one of the four instruments and leaves on these plants were selected randomly for gas exchange. Following established procedure (Long and Bernacchi 2003), each leaf was first allowed to achieve steady-state CO_2_ and water vapor exchange. The CO_2_ was then reduced stepwise to 50 *μ*mol·mol^−1^, returned to 400 *μ*mol·mol^−1^, and then increased stepwise to 1600 *μ*mol·mol^−1^. Each individual curve consisted of twelve separate measurements and took approximately 45 min to complete. Following completion of each curve, the leaf protruding from the leaf chamber was marked at the edge of the gasket. The leaf was then removed from the chamber and traced to allow determination of the leaf area enclosed by the chamber. Data were then recomputed using the measured leaf area. The photosynthetic parameters *V*_c,max_ and *J*_max_ were estimated as described previously (Bernacchi et al. 2006).

Glass microscope slides (2.5 cm × 7.6 cm) with adhesive were used to collect seeds. One slide was positioned per mesocosm prior to seed dispersal. Collected seeds were spread onto white paper, photographed, and digital images processed for seed number using ImageJ 1.41 software (Schneider et al. 2012).

Genotypic frequencies of mixed mesocosms were determined by randomly identifying and collecting a single leaf from 100 individuals. Each leaf was pressed into FTA paper (Whatman, GE Healthcare Life Sciences, Little Chalfont, England). Two punches (ea. 0.25 cm^2^) from the FTA paper were collected and used directly for PCR amplification of *nia2* boarder regions as per manufacturer directions using primers (right border; 5′ GATTTTTCGAGGTGACGTTCC 3′, left border; 5′ GTGGCCGGATGGTTAAATG 3′, and TLBa-1; 5′ TGGTTCACGTAGTGGGCCATCG 3′).

### Statistical analysis

Construction of the weighted gene co-expression network has been described previously (Zhang and Horvath 2005; Langfelder and Horvath 2008; Weston et al. 2008). The input microarray data were restricted to genes that were differentially expressed determined using the limma package to fit a linear model with CO_2_ and *nia2* mutant perturbations as contrasts and empirical Bayes to compute a moderated t-statistic according to Smyth (Smyth 2005). The above differential gene expression analysis identified 2767 genes that were entered into network algorithms. Weighted gene coexpression network (WGCNA) consists of four steps: (1) a pairwise Pearson correlation matrix created for all genes across all treatments; (2) transformation of correlations to connection strengths (connectivity) using a signed power adjacency function; (3) identification of modules or groups of highly correlated gene expression patterns by coupling linkage hierarchical clustering with topological overlap matrix; and (4) relating external gene or treatment information to network properties. In our case, the external treatment information was genotype and CO_2_.

Gene enrichment analysis was conducted using the classification SuperViewer Tool with bootstrap from the Povart Lab (http://bar.utoronto.ca/welcome.htm) that was linked to the MapMan classification source. The MapMan classification was downloaded from the TAIR10 on Aug 2012 (http://mapman.gabipd.org/web/guest/mapmanstore (file Ath_AGI_LOCUS_TAIR10_Aug2012.txt).

We used analysis of variance (ANOVA) to test whether CO_2_ and genotype affected measured metabolites, photosynthetic parameters, enzyme activities, and growth characteristics. For these analyses, all tests were conducted within generation as the sampling strategy for molecular data was collected from generations 2 and 4. Genotype, CO_2_, and their interactions were included as fixed factors, and significance was determined using *F*-tests.

To compare the consequences of the *nia2* mutant across levels of organization from biochemistry and gene networks to leaf-level physiology and population productivity, we calculated effect size using the log response ratio (ln *R*) (Souza et al. 2011):

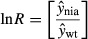
where, 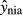 is the mean response variable for the *nia2* mutant genotype and 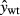 is the mean response variable for the wild-type genotype. All effect sizes using the above equation were performed on the current CO_2_ treatment, as CO_2_ was not significant for the response variables tested.

## Results

**Figure 1:**
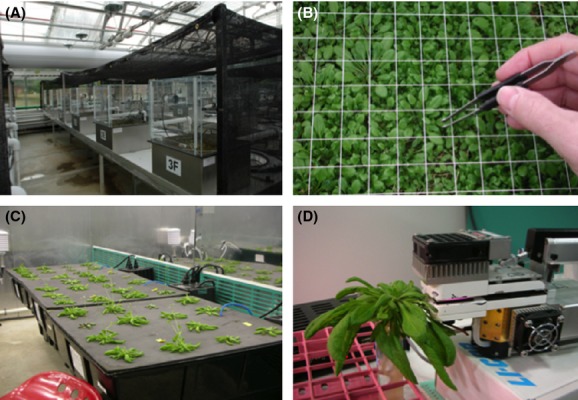
Two complementary approaches were used to examine the concept of robustness in wild-type and *nia2* mutants of Arabidopsis. Soil-based mesocosms (A) allowed sampling of plants (B) over multiple generations. Similarly, hydroponic mesocosms in growth chambers (C) facilitated measurements of plant biochemistry and photosynthesis (D) at current (400 ppm) and elevated CO_2_ (800 ppm) concentrations.

**Figure 2:**
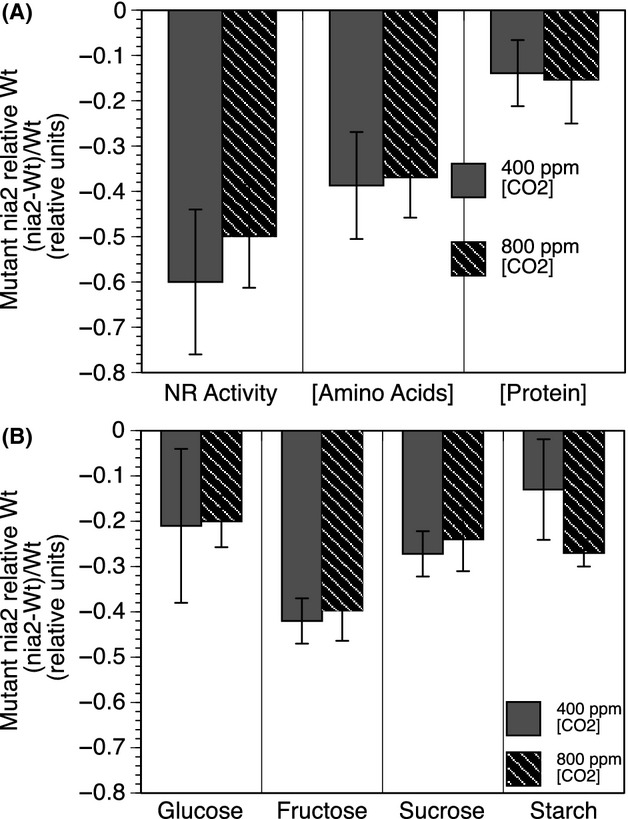
Relative difference between *nia2* mutant and wild-type (Wt) mesocosm populations in response to CO_2_. Consequences of genotype and CO_2_ on measured nitrate reductase enzyme activity, total amino acid, and soluble protein concentrations (A) and on selected reducing sugars and starch (B). Bars represent the standard mean estimates and standard error of the mean (SE) between 2 generations for (A). In (B), standard mean estimates and SE are specific to one generation (generation 2) as differences between generations were significant. ANOVA results are presented in Table1.

Plants were grown in a mesocosm environment with a constructed soil substratum (Fig.1A and B). The activity of the gateway enzyme in N metabolism, that is, nitrate reductase (*nia2*), was significantly reduced by the T-DNA insertion for both generations (Table1; Fig.2), with an average decline of 58% (SE = 14%) pooled over both generations relative to wild-type activity levels under current CO_2_. The *nia2* mutant affect cascaded to influence downstream N metabolism as observed by a 38% (SE = 6) decrease in total amino acids and a 13.9% (SE = 9) decrease in total protein relative to wild type (Fig.2). Genotype was significant for enzyme activity, total amino acid, and protein concentration at each generation, while CO_2_ was not (Table1). In general, carbon metabolism was influenced more by CO_2_ than by genotype. The storage carbohydrate starch was lower in the mutant relative to wild type, but was only significant (*P ≤ *0.05) for CO_2_ and not genotype in generation 2, while neither effect was significant in generation 4 (Table1). However, the consequences of CO_2_ and genotype on measured sugars (i.e., glucose, fructose, and sucrose) were more sporadic and not consistent between generations (Table1). Selected metabolites corresponding to C and N metabolism showed relatively minor differences between mutant and wild-type lines under the measured mesocosm conditions. Those that were significantly effected for both generations include the TCA-cycle intermediates citrate, fumarate, and malate. Galactose, glucose, and fructose were significantly different for both generations for CO_2_ but not genotype (Fig. S1).

**Table 1:** ANOVA results showing the effects of genotype, CO_2_, and their interactions on selected C and N metabolism intermediates. Presented are the *F* ratio and resulting *P*–value for the *F*-test in parentheses. All tests were performed within generation

	Generation 2			Generation 4		
	CO_2_	Genotype (G)	CO_2_ × G	CO_2_	Genotype (G)	CO_2_ × G
Nitrogen metabolism						
Nitrate reductase	2.28 (0.165)	42.71 (0.001)	1.48 (0.253)	1.05 (0.326)	96.54 (0.001)	7.73 (0.017)
Total amino acids	2.48 (0.148)	42.35 (0.001)	0.03 (0.847)	1.45 (0.253)	113.50 (0.001)	2.03 (0.181)
Total protein	3.03 (0.102)	44.54 (0.001)	0 (1)	0.01 (0.899)	62.54 (0.001)	0.01 (0.967)
Carbon metabolism						
Sucrose	2.20 (0.163)	0.21 (0.649)	4.71 (0.050)	5.27 (0.040)	0.03 (0.854)	0.07 (0.790)
Glucose	13.08 (0.010)	2.35 (0.150)	6.22 (0.028)	3.68 (0.079)	2.21 (0.162)	0.01 (0.925)
Fructose	11.01 (0.006)	20.93 (0.001)	1.12 (0.309)	2.10 (0.172)	12.82 (0.003)	0.52 (0.485)
Starch	21.39 (0.001)	0.22 (0.647)	0.11 (0.743)	0.03 (0.851)	0.04 (0.837)	0.14 (0.712)

**Figure 3:**
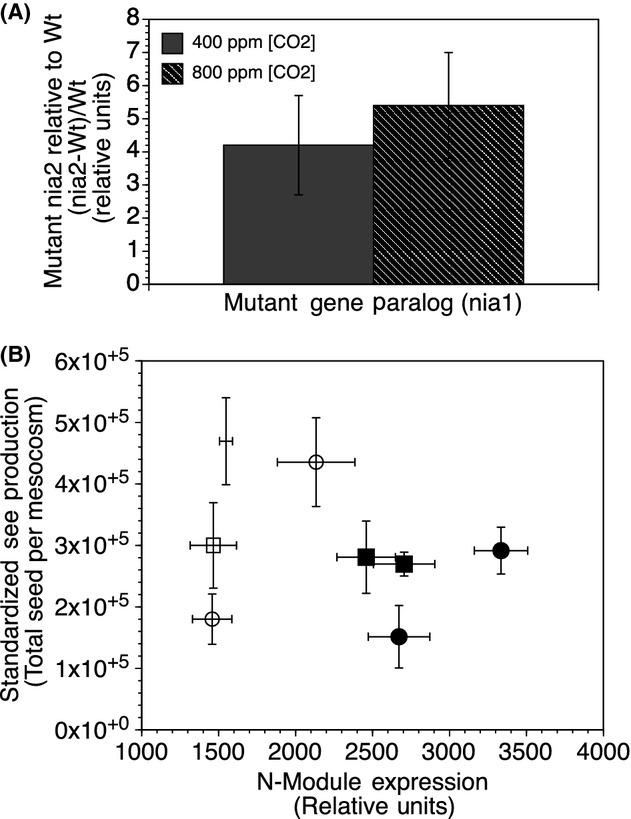
Relative difference in network module expression and seed production. Consequences of genotype and CO_2_ on the gene paralog to the mutant, *nia1* (A) relationship between the expression of the N network module and seed production (B). Bars represent the standard mean estimates and standard error of the mean (SE) between 2 generations for (A). In (B), clear symbols represent wild type and solid symbols are mutant. Circles are ambient, while squares are elevated CO_2_. Both generations 2 and 4 are graphed in B.

Because the consequences of the *nia2* mutation were more severe at the beginning of the nitrogen pathway (i.e., enzyme activity) relative to the end of the pathway (i.e., amino acid levels), we were motivated to investigate the mechanisms whereby molecular buffering occurred. At the gene expression level, the paralog to *nia2*, *nia1* (At1g77760) was significantly induced relative to wild type by ∼fourfold at current CO_2_ (*P *=* *1.8 × 10^−5^) and ∼sixfold at current CO_2_ (*P *=* *2.9 × 10^−4^) (Fig.3A), although CO_2_ was not significant as a main effect (*P *=* *0.146).

A gene co-expression network was constructed to investigate how single genes act in a coordinated fashion to the genetic and CO_2_ perturbations, resulting in six distinct groups of highly regulated genes termed modules (Fig. S2). Gene functional enrichment analysis revealed that the purple module was enriched with genes contributing to nitrogen metabolism and sulfur assimilation (Fig. S2) and was induced by 63% in generation 2 and 157% in generation 4 for the mutant population relative to the wild type. The eigenvalue for this module was significantly correlated to the *nia2* mutant (*r* = 0.82, *P *=* *0.001) and included genes connected within the module (i.e., hub genes) nitrate reductase 1 (*nia1*), nitrite reductase 1 (*nir1*, At2g15620), the nitrogen transporters NRT1.1 (At1g12110) and NRT 1.7 (At1g69870), and oligopeptide and amino acid transporters At4g10770 and At3g54830. The induction of the N module for the mutant population was associated with reduced fitness, as suggested by a 32% decrease in total seed production relative to the wild-type population (Fig.3B, *P* = 0.12).

The consequence of the *nia2* mutant on photosynthesis was evaluated using gas exchange. Photosynthesis (*A*) and the response of *A* to internal CO_2_ concentration (*c*_*i*_), commonly referred to as an *A*-*c*_*i*_ curve was used to measure the maximum capacity for carboxylation (*V*_c,max_) and the maximum rate of electron transport (*J*_max_). Because this measurement is impossible on the small leaves produced under mesocosm growth conditions, we grew the plants in growth chambers under conditions that produced leaves large enough for gas exchange measurements (hydroponics and a long photoperiod) but that also mimicked the treatments growth at eCO_2_ stimulated *A* as expected, but there was no effect of eCO_2_ on *V*_c,max_ or J_max_ (Table2). The Wild type had greater CO_2_ assimilation rates relative to the *nia2* mutant (Fig. S3A, Table2). This reduction in *A* is attributable to a 27% reduction in *V*_c,max_ and an 18% reduction in *J*_max_. No trends were apparent for differences in stomatal conductance (Fig. S3B) during the *A-C*_*i*_ curves.

**Table 2:** ANOVA table for net photosynthesis and photosynthetic parameters. Presented are the effects of the genotype, CO_2_, and their interaction on mean parameter value, standard error in parentheses, and *P*-value for the resulting *F*-test

	Mean (standard error)		*P*-value		
	Wt	*nia2*	Genotype (G)	CO_2_	CO_2_ × G
*V*_c,max_ (*μ*mol·CO_2_·m^−2^·sec^−1^)	72.7 (4.4)	53.13 (4.4)	0.036	0.420	0.754
*J*_max_ (*μ*mol·m^−2^·sec^−1^)	93.97 (3.12)	77.74 (3.12)	0.021	0.784	0.242
A_400_ (*μ*mol·CO_2_·m^−2^·sec^−1^)	9.54 (0.67)	6.98 (0.67)	0.056	0.607	0.806
A_800_ (*μ*mol·CO_2_·m^−2^·sec^−1^)	16.41 (0.895)	12.88 (0.895)	0.049	0.802	0.440

**Figure 4:**
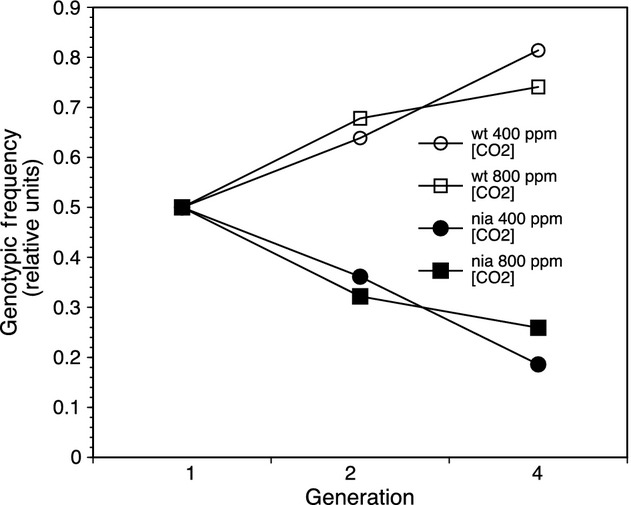
Population dynamics through generation. Clear symbols represent wild type and solid symbols are mutant. Circles are current 400 ppm [CO_2_], while squares are elevated CO_2_ at 400 ppm [CO_2_]. Both generations 2 and 4 are graphed in B.

**Figure 5:**
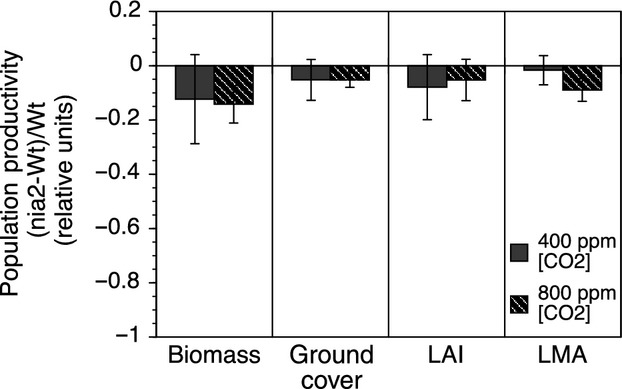
Relative difference among population productivity estimates. Relative consequences of genotype and CO_2_ on population-level biomass, ground cover, leaf area index (LAI), and leaf mass per area (LMA). Error bars denote the standard error of the mean.

**Figure 6:**
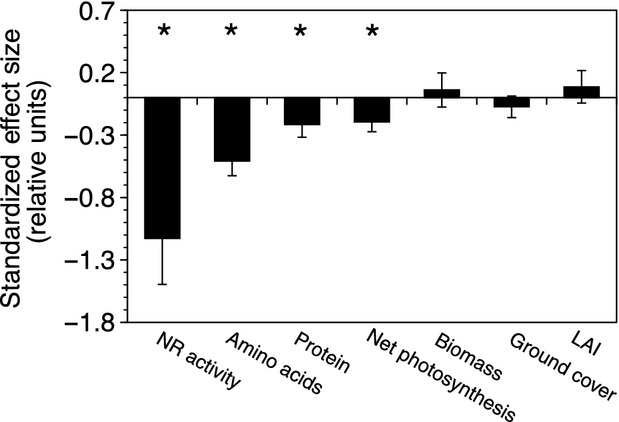
Consequences of genetic perturbation through levels of biological organization. Standardized effect size through molecular to organismal and population levels. Error bars denote the standard error of the mean effect size and the presence of asterisks denotes that the value is significantly different from 0 at *P* < 0.05.

Population dynamics in the mixed mesocosms changed over the course of the experiment by increasing wild-type genotypic frequency from 50% to 85% over (Fig.4). However, mesocosm productivity changes, as estimated by aboveground biomass, leaf area index, leaf mass per area, and total seed production, were relatively minor (Fig.5). An evaluation of the effect size for the single-gene mutation from N-activity, to individual leaf-level physiology to population productivity showed a decreasing trend through biological organization (Fig.6).

## Discussion

In our simplified model ecosystem, we found that the disproportionate investment by the mutant population in the induction of *nia1* and induction of the N-assimilation network module was used to buffer against the genetic perturbation in *nia2*. These buffering mechanisms likely resulted in a physiologic and metabolic cost that lowered mutant photosynthesis and fitness, shifted population dynamics and slightly altered population function. These results are in support of our first hypothesis where a single-gene effect cascaded to alter individual plant function. However, we must reject our second hypothesis, as we found no significant differences for eCO_2_ in modifying the consequences of this genetic perturbation to population dynamics or function. We show that the consequence of the genetic perturbation is reduced through levels of biological organization presumably through the expression of buffering mechanisms. A discussion of those buffering mechanisms and their consequences on organismal fitness and population function are discussed below.

### Potential buffering mechanisms at the molecular level

Standardized effect size analysis showed that the negative consequences from the single-gene mutant at the *nia2* locus had a marked effect at the start of the pathway (i.e., nitrate reductase activity) that gradually decreased by the end of the pathway (i.e., amino acid levels). Our analyses indicated that a number of compensation reactions were induced in response to the genetic perturbation and thereby provide a glimpse into the mechanisms used to maintain phenotypic stability in the face of environmental, stochastic, and genetic variations often referred to as biological buffering or robustness (Hartman et al. 2001).

Our gene expression analysis indicated that the gene paralog to the mutant, *nia1,* was greatly induced. For wild-type Arabidopsis seedlings, *nia2* is roughly responsible for 90% of the nitrate reductase activity, while *nia1* accounts for the remaining 10% (Wilkinson and Crawford 1991). Although the tissue-specific transcriptional responses of *nia1* and *nia2* can differ under some conditions including light (Cheng et al. 1991) and cytokinin application (Yu et al. 1998), the *nia2* deletion mutant can grow normally on nitrate-containing medium based on *nia1* activity alone (Wilkinson and Crawford 1993). Theoretical studies suggest that functional compensation by gene duplicates can be evolutionarily preserved and experimental evidence in support of this theory has been reported for a number of organisms including yeast (Wagner 2000), nematodes (Conant and Wagner 2004), and Arabidopsis (Hanada et al. 2009) and has been shown to be a key mechanism by which primary and secondary metabolism is preserved for *Arabidopsis thaliana* (Hanada et al. 2011).

We used gene co-expression network analysis and targeted metabolic profiles to provide a more holistic view of genomic reprogramming associated with the genetic and CO_2_ perturbations. Our network analysis identified a group of highly correlated genes, termed modules, that was associated with the mutant. This N-responsive module was induced in the mutant relative to the wild-type and contained known N-responsive genes including *nia1* (discussed above) and the nitrate transporter encoding gene *NRT1.1* as hub genes within the network (highly connected node). Both genes have previously been shown to be induced within *nia2* deletion plants (Loque et al. 2003). The network analysis also revealed that the same N module was enriched with genes functioning in sulfur assimilation. Sulfate reduction must compete with N assimilation for reducing equivalents and previous studies have reported reduced maximal extractable nitrate reductase activity in sulfur-deprived tobacco plants (Migge et al. 2000), while sulfur addition has been shown to increase nitrate reductase activity (Jamal et al. 2006), thereby supporting the link between N and S metabolism. This systems’ level view provided by network analysis has been previously used to implicate buffering of yeast phenotypes to environmental variation (Levy and Siegal 2008) and was more recently applied to *Arabidopsis* knockouts (Wu and Qi 2010).

### Potential buffering mechanisms at the physiologic level

*nia2* mutants, like the one used within the current study, have been shown to provide an N-limitation phenotype even when supplied with nitrate (Loque et al. 2003) and such N-limiting conditions have been known to reduce photosynthesis for quite some time (Natr 1972). Here, we measured key photosynthetic parameters and found that maximal rates of Rubisco carboxylation (*V*_c,max_) and the maximal rates of electron transport (*J*_max_) were reduced for genotypes harboring the *nia2* mutant. However, our standardized effect analysis showed that the reduction in net photosynthesis followed the decreasing trend in effect size as we scaled across levels of biological organization, suggesting that further buffering mechanisms were at play. Although our research does not elude directly to any such mechanisms, we know that the N economy of the plant can be altered and hypothesize that N-limiting plants can modify allocation patterns to balance nitrogen investment for various biological pathways (Xu et al. 2012).

Plants exposed to eCO_2_ typically have reduced protein concentration relative to plants grown under ambient conditions. A number of hypotheses have been posed to explain this phenomenon including protein dilution as CO_2_ results in larger plants (Ellsworth et al. 2004), reduced Rubisco protein amount due to downregulation from carbohydrate accumulation (Long et al. 2004), progressive soil N limitation as the CO_2_ enriched plants deposit more carbon to rhizosphere (Reich et al. 2006a,b), and more recently there is increasing evidence that elevated CO_2_ directly inhibits plant 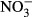 assimilation into protein (see review Bloom 2015). It is noteworthy that we did not see a significant reduction in tissue N for our CO_2_ treatment alone possibly due to soil N status, mesocosm heterogeneity, or accumulation of nitrate that was unable to be converted to nitrite. It is also possible that there where adjustments in growth are considered a key response of acclimation (Tocquin et al. 2006; Tschoep et al. 2009) and may represent a new homeostatic state for the plant.

### Genetic and CO_2_ perturbations at the population level

The relationship between N-module expression and total seed production was negatively correlated and may reflect the internal costs associated with the expression of the buffering mechanisms. This reduction in seed production translated into a consistent, yet fairly dramatic shift from mutant genotype to wild-type genotype within the mixed mesocosms through six generations. One of the most surprising aspects of this study was the relatively mild effect that this change in population dynamics had on population functions as estimated by mesocosm-level biomass, leaf area index, leaf mass per area, and total ground cover. In essence, the large effect seen from the single-gene mutation on nitrate reductase activity was nearly negligible as we scaled to the population level. This illustrates the challenge in scaling mechanistic information that may be informative for predicting organismal traits to how those traits will influence growth within a competitive environment. At the population level, for example, canopy light interception may be more positively associated with productivity than leaf-level traits for some systems (e.g., McCrady and Jokela 1996; Chmura and Tjoelker 2008).

## Conclusions

A number of studies have shown that single-gene mutant do not always result in a noticeable phenotype as a result of multiple biological buffering mechanisms ranging from partial redundancy in gene paralogs and phenotypic capacitors to network architecture. In the current study, we show that a single-gene mutation in the gateway enzyme to the N-assimilation pathway is followed by effects that became increasing less evident through levels of biological organization via buffering from gene paralogs, changes in network architecture, and mechanisms yet to be discovered. The manifestation of these mechanisms may have resulted in metabolic costs that were expressed as reductions in net photosynthesis, fitness, and changes in population dynamics. Nonetheless, the effect of the population change had a negligible effect on mesocosm productivity, highlighting the fact that buffering mechanisms exist at population and ecosystems levels (May 2006; Wilmers 2007).

The perception that buffering and robustness mechanisms are always beneficial may be overly simplistic, especially when viewed through an evolutionary framework. This was articulated by Frank (2007), who used a mathematical model to show that enhanced robustness can cause an evolutionary reduction in the adaptive performance of the target trait. The reduced selective pressure on the target trait can result in lower trait performance. Over evolutionary time, the layering of buffering mechanisms may result in lower adaptive trait performance resulting in the “paradox of robustness.” A future challenge will be in placing buffering mechanisms within a predictive and evolutionary framework across levels of biological organization from cells to ecosystems.

## Data Accessibility

All gene expression data are publically available at the NCBI Gene Expression Omnibus database (http://www.ncbi.nlm.nih.gov/geo/) as accession GSE16068. Construction of the gene co-expression network was generated using publically availably R scripts (refer to Materials & Methods) and network results.

## Supporting Information

**Figure S1.** Relative difference in metabolites between *nia2* mutant and wild-type (Wt) mesocosm populations in response to CO_2_. **Figure S2.** Multiple dimensional scaling plot of the co-expression network nodes and enrichment analysis. **Figure S3.** Response of net photosynthesis and stomatal conductance on internal CO_2_.

## References

[b1] Ainsworth EA, Rogers A, Leakey AD, Heady LE, Gibon Y, Stitt M (2007). Does elevated atmospheric [CO_2_] alter diurnal C uptake and the balance of C and N metabolites in growing and fully expanded soybean leaves?. J. Exp. Bot.

[b2] Bantan-Polak T, Kassai M, Grant KB (2001). A comparison of fluorescamine and naphthalene-2,3-dicarboxaldehyde fluorogenic reagents for microplate-based detection of amino acids. Anal. Biochem.

[b3] Bartholomew GA, Hg M (1964). The roles of physiology and behaviour in the maintenance of homeostasis in the desert environment. Homeostasis and feedback mechanisms.

[b4] Bernacchi CJ, Morgan PB, Ort DR, Long SP (2005). The growth of soybean under free air [CO_2_] enrichment (FACE) stimulates photosynthesis while decreasing in vivo Rubisco capacity. Planta.

[b5] Bernacchi CJ, Leakey ADB, Heady LE, Morgan PB, Dohleman FG, McGrath JM (2006). Hourly and seasonal variation in photosynthesis and stomatal conductance of soybean grown at future CO_2_ and ozone concentrations for 3 years under fully open-air field conditions. Plant, Cell Environ.

[b6] Bloom AJ (2015). Photorespiration and nitrate assimilation: a major intersection between plant carbon and nitrogen. Photosynth. Res.

[b7] Chapin FS, Schulze ED, Mooney HA (1990). The ecology and economics of storage in plants. Annu. Rev. Ecol. Syst.

[b8] Cheng CL, Acedo GN, Dewdney J, Goodman HM, Conkling MA (1991). Differential expression of the two *Arabidopsis* nitrate reductase genes. Plant Physiol.

[b9] Chmura DJ, Tjoelker MG (2008). Leaf traits in relation to crown development, light interception and growth of elite families of loblolly and slash pine. Tree Physiol.

[b10] Conant GC, Wagner A (2004). Duplicate genes and robustness to transient gene knock-downs in *Caenorhabditis elegans*. Proc. Biol. Sci.

[b11] Delattre M, Felix MA (2009). The evolutionary context of robust and redundant cell biological mechanisms. BioEssays.

[b12] Dunne JA, Williams RJ, Martinez ND (2002). Network structure and biodiversity loss in food webs: robustness increases with connectance. Ecol. Lett.

[b13] Ellsworth DS, Reich PB, Naumburg ES, Koch GW, Kubiske ME, Smith SD (2004). Photosynthesis, carboxylation and leaf nitrogen responses of 16 species to elevated pCO_2_ across four free-air CO_2_ enrichment experiments in forest, grassland and desert. Glob. Change Biol.

[b14] Evans JR, Poorter H (2001). Photosynthetic acclimation of plants to growth irradiance: the relative importance of specific leaf area and nitrogen partitioning in maximizing carbon gain. Plant, Cell Environ.

[b15] Frank SA (2007). Maladaptation and the paradox of robustness in evolution. PLoS One.

[b16] Gibon Y, Blaesing OE, Hannemann J, Carillo P, Höhne M, Hendriks JH (2004). A robot-based platform to measure multiple enzyme activities in *Arabidopsis* using a set of cycling assays: comparison of changes of enzyme activities and transcript levels during diurnal cycles and in prolonged darkness. Plant Cell.

[b17] Gillespie KM, Rogers A, Ainsworth EA (2011). Growth at elevated ozone or elevated carbon dioxide concentration alters antioxidant capacity and response to acute oxidative stress in soybean (*Glycine max*. J. Exp. Bot.

[b19] Greenbury SF, Johnston IG, Smith MA, Doye JPK, Louis AA (2010). The effect of scale-free topology on the robustness and evolvability of genetic regulatory networks. J. Theor. Biol.

[b20] Gutierrez RA, Lejay LV, Dean A, Chiaromonte F, Shasha DE, Coruzzi GM (2007). Qualitative network models and genome-wide expression data define carbon/nitrogen-responsive molecular machines in *Arabidopsis*. Genome Biol.

[b21] Hanada K, Kuromori T, Myouga F, Toyoda T, Wen-Hsiung L, Shinozaki K (2009). Evolutionary persistence of functional compensation by duplicate genes in *Arabidopsis*. Genome Biol. Evol.

[b22] Hanada K, Sawada Y, Kuromori T, Klausnitzer R, Saito K, Toyoda T (2011). Functional compensation of primary and secondary metabolites by duplicate genes in *Arabidopsis thaliana*. Mol. Biol. Evol.

[b23] Hartman JL, Garvik B, Hartwell L (2001). Cell biology - principles for the buffering of genetic variation. Science.

[b24] Hoff T, Truong HN, Caboche M (1994). The use of mutants and transgenic plants to study nitrate assimilation. Plant, Cell Environ.

[b25] Jamal A, Fazli IS, Ahmad S, Abdin MZ, Yun SJ (2006). Effect of sulfur on nitrate reductase and ATP sulfurylase activities in groundnut (*Arachis hypogea* L.). J. Plant Biol.

[b26] Kinugasa T, Hikosaka K, Hirose T (2003). Reproductive allocation of an annual, *Xanthium canadense*, at an elevated carbon dioxide concentration. Oecologia.

[b28] Langfelder P, Horvath S (2008). WGCNA: an R package for weighted correlation network analysis. BMC Bioinformatics.

[b29] Lejay L, Tillard P, Lepetit M, Olive FD, Filleur S, Daniel-Vedele F (1999). Molecular and functional regulation of two NO_3_^−^ uptake systems by N- and C-status of *Arabidopsis* plants. Plant J.

[b30] Levy SF, Siegal ML (2008). Network hubs buffer environmental variation in *Saccharomyces cerevisiae*. PLoS Biol.

[b31] Long SP, Bernacchi CJ (2003). Gas exchange measurements, what can they tell us about the underlying limitations to photosynthesis? Procedures and sources of error. J. Exp. Bot.

[b32] Long SP, Ainsworth EA, Rogers A, Ort DR (2004). Rising atmospheric carbon dioxide: plants face the future. Annu. Rev. Plant Biol.

[b33] Loque D, Tillard P, Gojon A, Lepetit M (2003). Gene expression of the NO_3_ transporter NRT1.1 and the nitrate reductase *nia1* is repressed in *Arabidopsis* roots by NO_2_^−^, the product of NO_3_ reduction. Plant Physiol.

[b34] Masel J, Siegal ML (2009). Robustness: mechanisms and consequences. Trends Genet.

[b35] May RM (2006). Network structure and the biology of populations. Trends Ecol. Evol.

[b36] May P, Liao W, Wu Y, Shuai B, McCombie WR, Zhang MQ (2013). The effects of carbon dioxide and temperature on microRNA expression in *Arabidopsis* development. Nat. Commun.

[b37] McCrady RL, Jokela EJ (1996). Growth phenology and crown structure of selected loblolly pine families planted at two spacings. For. Sci.

[b38] Melian CJ, Bascompte J (2002). Complex networks: two ways to be robust?. Ecol. Lett.

[b39] Migge A, Bork C, Hell R, Becker TW (2000). Negative regulation of nitrate reductase gene expression by glutamine or asparagine accumulating in leaves of sulfur-deprived tobacco. Planta.

[b40] Morse AM, Tschaplinski TJ, DerviniS C, Pijut PM, Schmelz EA, Day W (2007). Salicylate and catechol levels are maintained in nahG transgenic poplar. Phytochemistry.

[b41] Natr L (1972). Influence of mineral nutrients on photosynthesis of higher plants. Photosynthetica.

[b42] Norby RJ, Wullschleger SD, Gunderson CA, Johnson DW, Ceulemans R (1999). Tree responses to rising CO_2_ in field experiments: implications for the future forest. Plant, Cell Environ.

[b43] Noren H, Svensson P, Andersson B (2004). A convenient and versatile hydroponic cultivation system for *Arabidopsis thaliana*. Physiol. Plant.

[b44] Nunes-Nesi A, Fernie AR, Stitt M (2010). Metabolic and signaling aspects underpinning the regulation of plant carbon nitrogen interactions. Mol. Plant.

[b45] Pritchard SG, Rogers HH, Prior SA, Peterson CM (1999). Elevated CO_2_ and plant structure: a review. Glob. Change Biol.

[b46] Reich PB, Hungate BA, Luo YQ (2006a). Carbon-nitrogen interactions in terrestrial ecosystems in response to rising atmospheric carbon dioxide. Annu. Rev. Ecol. Evol. Syst.

[b47] Reich PB, Hobbie SE, Lee T, Ellsworth DS, West JB, Tilman D (2006b). Nitrogen limitation constrains sustainability of ecosystem response to CO_2_. Nature.

[b48] Reusch TB, Wood TE (2007). Molecular ecology of global change. Mol. Ecol.

[b49] Sage RF, Pearcy RW, Seemann JR (1987). The nitrogen use efficiency of C(3) and C(4) plants: III. Leaf nitrogen effects on the activity of carboxylating enzymes in *Chenopodium album* (L.) and *Amaranthus retroflexus* (L.). Plant Physiol.

[b500] Schneider CA, Rasband WS, Eliceiri KW (2012). NIH Image to ImageJ: 25 years of image analysis. Nat. Methods.

[b50] Smyth GK, Gentleman R, Carey V, Dudoit S, Irizarry R, Huber W (2005). Limma: linear models for microarray data. Bioinformatics and computational biology solutions using R and bioconductor.

[b51] Souza L, Weston DJ, Sanders NJ, Karve A, Crutsinger GM, Classen AT (2011). Intraspecific variation in response to warming across levels of organization: a test with *Solidago altissima*. Ecosphere.

[b52] Tocquin P, Ormenese S, Pieltain A, Detry N, Bernier G, Périlleux C (2006). Acclimation of *Arabidopsis thaliana* to long-term CO_2_ enrichment and nitrogen supply is basically a matter of growth rate adjustment. Physiol. Plant.

[b53] Tschoep H, Gibon Y, Carillo P, Armengaud P, Szecowka M, Nunes-Nesi A (2009). Adjustment of growth and central metabolism to a mild but sustained nitrogen-limitation in *Arabidopsis*. Plant Cell Environ.

[b54] Wagner A (2000). Robustness against mutations in genetic networks of yeast. Nat. Genet.

[b55] Wang R, Okamoto M, Xing X, Crawford NM (2003). Microarray analysis of the nitrate response in *Arabidopsis* roots and shoots reveals over 1000 rapidly responding genes and new linkages to glucose, trehalose-6-phosphate, iron, and sulfate metabolism. Plant Physiol.

[b56] Weston DJ, Gunter LE, Rogers A, Wullschleger SD (2008). Connecting genes, coexpression modules, and molecular signatures to environmental stress phenotypes in plants. BMC Syst. Biol.

[b57] Weston DJ, Karve AA, Gunter LE, Jawdy SA, Yang X, Allen SM (2011). Comparative physiology and transcriptional networks underlying the heat shock response in *Populus trichocarpa Arabidopsis thaliana* and *Glycine max*. Plant, Cell Environ.

[b58] Wilkinson JQ, Crawford NM (1991). Identification of the *Arabidopsis chl3* gene as the nitrate reductase structural gene *nia2*. Plant Cell.

[b59] Wilkinson JQ, Crawford NM (1993). Identification and characterization of a chlorate-resistant mutant of *Arabidopsis thaliana* with mutations in both nitrate reductase structural genes *nia1* and *nia2*. Mol. Gen. Genet.

[b60] Wilmers CC (2007). Understanding ecosystem robustness. Trends Ecol. Evol.

[b61] Wu X, Qi X (2010). Genes encoding hub and bottleneck enzymes of the *Arabidopsis* metabolic network preferentially retain homeologs through whole genome duplication. BMC Evol. Biol.

[b62] Xu C, Fisher R, Wullschleger SD, Wilson CJ, Cai M, McDowell NG (2012). Toward a mechanistic modeling of nitrogen limitation on vegetation dynamics. PLoS One.

[b63] Yu X, Sukumaran S, Mrton L (1998). Differential expression of the *Arabidopsis nia1* and *nia2* genes. cytokinin-induced nitrate reductase activity is correlated with increased *nia1* transcription and mRNA levels. Plant Physiol.

[b64] Zhang B, Horvath S (2005). A general framework for weighted gene co-expression network analysis. Stat. Appl. Genet. Mol. Biol.

