# Supplementary material for: Scaling nitrogen and carbon interactions: what are the consequences of biological buffering?
Source: Ecol Evol. 2015 Jun 25;5(14):2839–50. doi: 10.1002/ece3.1565 (PMC4541989; doi:10.1002/ece3.1565)
Supplement: Supplementary file 1 [file ece30005-2839-sd1.docx]

**ECE_ ECE-2015-05-00295**

**Supplemental information:**

**Supplemental Fig. S1**

**Fig. S1. Relative difference in metabolites between *nia2* mutant and wild-type (Wt) mesocosm populations in response to CO_2_**. All generations for selected metabolites at ambient CO_2_ are presented. Symbols indicate the ratio for which the CO_2_ treatment was significant at *P*<0.001 (***), *P*<0.01 (**), *P*<0.05 (*).

**Fig. S2. Multiple dimensional scaling plot of the co-expression network nodes and enrichment analysis.** Each circle represents a single gene and the color of the circle corresponds to module designation, and is independent of the colors in (B).The distance between circles is a function of topological overlap and provides a visual representation of gene and module relationships within network (A). Graphical results of gene enrichment analysis for the N – module only (B). The functional classification is based on the GO database and provided is a ranking score ( y – axis) calculated for each functional class, and error bars provide some idea as to over- or under-representation reliability based on a bootstrapped set of 100 times.

**Supplemenatl Fig. S2**


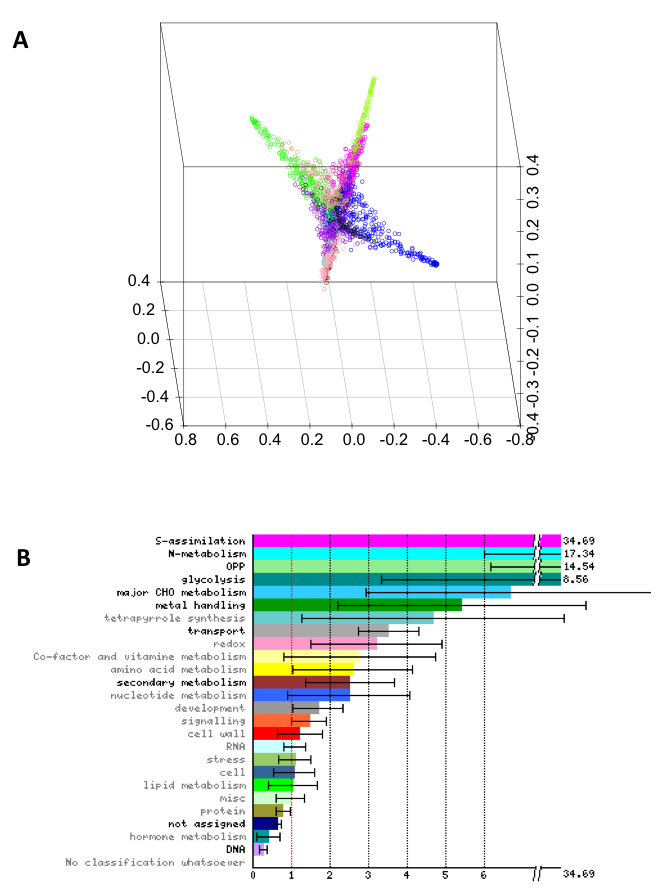


**Supplemental Fig. S3**

**Fig. S3. Response of net photosynthesis and stomatal conductance on internal CO_2_.** Response of net photosynthesis (A) and Stomatal conductance (B) to internal CO_2_ conditions (*C_i_*). Solid circles are observations from *nia2* mutants while open circles are from wild-type plants.
